# Initial subjective reward: single-exposure conditioned place preference to alcohol in mice

**DOI:** 10.3389/fnins.2014.00345

**Published:** 2014-11-04

**Authors:** Judith E. Grisel, John B. Beasley, Emma C. Bertram, Brooke E. Decker, Chunyu A. Duan, Mahder Etuma, Annie Hand, Mallory N. Locklear, Matthew P. Whitmire

**Affiliations:** ^1^Psychology, Bucknell UniversityLewisburg, PA, USA; ^2^Neuroscience, Furman UniversityGreenville, SC, USA; ^3^Neuroscience, Bucknell UniversityLewisburg, PA, USA; ^4^Princeton Neuroscience Institute, Princeton UniversityPrinceton, NJ, USA; ^5^Neurology and Behavior, Stony Brook University, State University of New YorkStony Brook, NY, USA

**Keywords:** addiction, vulnerability, subjective reward value, animal models of mental disorders, translational research

## Abstract

Most adults consume alcohol with relative impunity, but about 10–20% of users persist (or progress) in their consumption, despite mounting and serious repercussions. Identifying at-risk individuals before neuroadaptative changes associated with chronic use become well ingrained is thus a key step in mitigating and preventing the end stage disease and its devastating impacts. Explaining liability has been impeded, in part, by the absence of animal models for assessing initial sensitivity to the drug's reinforcing properties, an important endophenotype in the trajectory toward excessive drinking. Here we assess the initial rewarding effects of the drug in a novel application of the conditioned place preference paradigm. In contrast to previous studies that have all employed repeated drug administration, we demonstrated a robust preference for a context paired with a single exposure to 1.5 g/kg EtOH in male and female subjects of three strains. This model validates an assay of initial sensitivity to the subjective rewarding effects of alcohol, a widely used drug with multifarious impacts on both brain and society, and provides a new tool for theory-driven endophenotypic pharmacogenetic approaches to understanding and treating addiction.

## Introduction

Though moderate alcohol use is widespread, a portion of users accrue a range of serious adverse consequences, yet maintain excessive consumption (World Health Organization, 2011; Moss et al., 2012; Rehm et al., 2014). In the face of a growing imbalance between costs and benefits of drinking, an alcoholic's irrational choices inevitably wreak havoc in the lives of family members, impair or destroy personal relationships, and compromise workplace activities and personal health.

Despite decades of basic research, and known heritability (Reich et al., 1998; Prescott and Kendler, 1999; Enoch, 2013) neural antecedents for this disease remain obscure. The gap in understanding reflects the fact that, like other heterogeneous behavioral traits, the tendency toward excessive drinking is mediated by interactions between a biological predisposition—largely driven by common variants in many genes—with a multitude of developmental and other environmental influences (Ducci and Goldman, 2012). Progress thus rests on parsing the broad clinical complexity into more narrowly defined endophenotypes that are intermediate in the chain of causality from genes to disease (Porjesz and Begleiter, 1998; Burmeister, 1999; Seong et al., 2002; Gould and Gottesman, 2006; Crabbe, 2012). One such heritable, quantitative component is the pleasurable, subjective response to the drug, as it depends upon the drug's influence in multiple neural pathways, varies across the population, and predicts disordered drinking (Heath and Martin, 1991; Schuckit and Smith, 1996; Schuckit et al., 1996; Viken et al., 2003; Ray et al., 2010).

Delineating relevant endophenotypes within a diverse clinical population steers the development of appropriate animal models, which can play a critical role in elucidating underlying neural substrates (Gould and Gottesman, 2006; Camarini et al., 2010). At present, the field lacks an efficient model for assessing the initial subjective experience to alcohol, contributing to an exigency in basic studies of the *antecedent causes* of the disease, and an emphasis on evaluating *consequences* of repeated exposure. For instance, extant models of alcohol reinforcement, including self-administration, locomotor sensitization, and conditioned place preference (CPP) all employ multiple drug exposures, and therefore fail to isolate initial sensitivity to the rewarding effects of the drug (Cunningham et al., 2000; Stephens et al., 2010; Bell et al., 2012). The paucity of animal models for assessing innate liability is a major limitation to translating findings in genetics and molecular and cell biology to the clinic, where diagnosis is based on self-report or other observations of harm, again *following* excessive use, rather than on biomarkers that predict disordered drinking. Better understanding of the biologically vulnerable phenotype would be useful in designing more effective interventions and treatments.

Here we describe a new tool to facilitate experimental testing of particular genetic or neurochemical influences on the initial rewarding effects of alcohol. Our studies probe innate liability by assessing subjective reward (Wise and Bozarth, 1987; Newlin and Thomson, 1999; Ray et al., 2010, 2013) following the conceptual underpinnings of the classic conditioned place preference (CPP) paradigm. In this paradigm, pharmacological effects of a drug are associated with drug-paired environmental cues, and a behavioral preference for the drug-paired “place” over another associated with saline administration is used to draw inferences about the drug's subjective effects (Cunningham et al., 2006).

Our studies in mice provide evidence of robust place preference for a context that has been paired once with a moderate dose of alcohol (EtOH). In contrast to published investigations of EtOH CPP, this paradigm involves no habituation to the testing apparatus or experimenter handling, the dose of EtOH is relatively low (1.5 g/kg), and subjects demonstrate robust place preference after only a single drug exposure.

## Materials and methods

### Subjects

Mice were either bred in-house (C57BL/6) from stock obtained from Jackson Laboratory (Bar Harbor, ME), or shipped directly from Jackson Laboratory (DBA/2) or from Hilltop Laboratories (Scottdale, PA; Swiss Webster) at 6–7 weeks old. Male and female mice were included in all studies, in approximately equal numbers and tested between 8 and 12 weeks of age, in a protocol reviewed and approved by the Bucknell University Animal Care and Use Committee and in accordance with the National Institutes of Health guidelines for ethical and humane animal research.

The colony was maintained on a 12:12 reverse light-dark cycle (lights off at 09:30) in a temperature (22 ± 2°C) and humidity (50 ± 20%) controlled environment. After weaning or arriving from suppliers, mice were housed 2–5/cage in Plexiglas caging on Thoren racks, by sex. Water and food were available *ad libitum.* Cages were checked daily, but bedding was not changed during the 5-day experimental period which took place after at least 2 weeks of acclimation to the colony room, in the case of shipped subjects, and in 60–100 day old subjects.

### Conditioning apparatus

**Figure 1:**
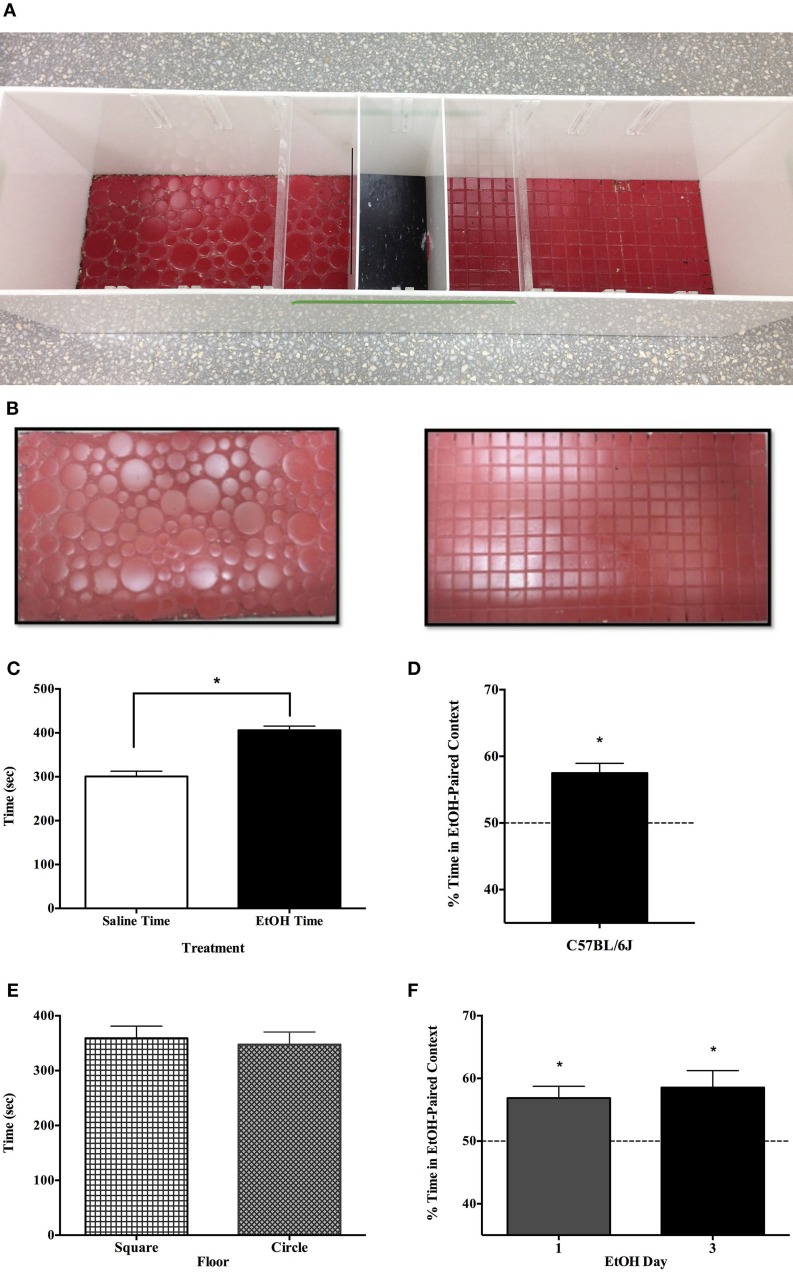
**Demonstration of single-exposure conditioned place preference to EtOH.** Each apparatus had three compartments including a neutral center compartment and two conditioning chambers that were distinguished by the pattern of tile floor **(A,B)**. All experiments were counterbalanced in terms of the drug-paired conditioning context (Circle or Square tile) and the day of EtOH exposure (day 1 or 3 of conditioning). After receiving 1.5 g/kg EtOH on day 1 or 3 of a 5-day protocol, adult C57BL/6J mice in Experiment 1a (*n* = 8) preferred the alcohol-paired context to the saline-paired context on day 5 **(C)**. The nearly 2 min difference between the time spent on the sides was significantly greater than zero [*t*_(7)_ = 5.282, *p* < 0.01]. **(D)** Subtracting out time spent in the neutral center chamber, indicates that on average, subjects spent about 57% of their onside-time in the EtOH-paired context, as opposed to about 43% in the saline-paired environment [*t*_(7)_ = 39.362, *p* < 0.001]. The experimental apparatus employed in these studies was unbiased, and subjects were equally likely to have EtOH on either floor (painted circle or square tile) and time spent in both contexts was equal on the test day; *t*_(7)_ = 0.260, *p* = 0.803 **(E)**. Finally, the day of EtOH administration did not affect CPP as subjects receiving EtOH on day 1 or 3 showed comparable preference for the EtOH-paired floor on day 5 [*F*_(1, 7)_ = 0.277, *p* = 62] **(F)**. ^*^indicates *p* < 0.05.

Our apparatus was unbiased, employing two distinct floor tile patterns available at our local home improvement store. One was comprised of circles of various sizes, and the other of uniform square tiles; both were painted the same color red (see Figure 1B). These floors (42 × 24 cm) served as conditioning contexts, in a 3 chamber apparatus (Figure 1A) that was otherwise opaque white Plexiglas. The center chamber was smaller than the others (11.5 × 24) and intended to be stimulus-neutral with a smooth black floor.

### Experimental protocol

#### Experiment 1a, 1b, and 1c

The experimental protocol was conducted over 5 days, including conditioning on days 1 and 3 and testing on day 5 (subjects were left undisturbed in home cages on days 2 and 4). The protocol was fully counterbalanced so that each animal received one injection of drug and one of saline in either of two distinct contexts, on either day 1 or 3 (See Table 1).

**Table 1:** **Depicts the 3 day protocol, occurring across 5 days, and demonstrates how day of EtOH administration and conditioning context were counterbalanced in all groups**.

**Experimental groups**	**Day 1**	**Day 3**	**Test day 5**
1	1.5 g/kg EtOH (20% in saline, delivered i.p.) on circle-floor	Saline on square-floor	Saline injection followed by free access to three compartments (one neutral, 2 conditioning; see Figure 1)
2	1.5 g/kg EtOH (20% in saline, delivered i.p.) on square-floor	Saline on circle-floor	
3	Saline on square-floor	1.5 g/kg EtOH (20% in saline, delivered i.p.) on circle-floor	Saline injection followed by free access to three compartments
4	Saline on circle-floor	1.5 g/kg EtOH (20% in saline, delivered i.p.) square-floor	

On days 2 and 4 mice were left undisturbed in their home cages.

On each of the three experimental days, subjects were removed from the colony one cage at a time and taken across the hall to the dimly lit testing suite where they were weighed and tail marked with colored marker. Intraperitoneal (i.p.) injections of either 1.5 g/kg EtOH (20% by volume in saline) or equivolume saline were administered 10–40 min after weighing and subjects were placed into one side of the conditioning apparatus. On conditioning days mice were relegated to either side compartment for 20–30 min, beginning immediately after injection. To avoid possible confound induced by differential social interaction subsequent to conditioning, all animals in each cage received the same injection.

On the test day the Plexiglas partitions between chambers were inverted, opening a 2″ port allowing free access between chambers, and subjects were placed in the center chamber immediately following saline (i.p.). The conditioning and testing periods varied slightly across experiments, ranging from 20 to 30 min. The apparatus was cleaned using a sponge filled with a dilute, low-residue detergent between each subject's exposure.

*Experiment 1a* employed 8 C57BL/6J mice (5 female and 3 male).

*Experiment 1b* replicated the protocol of Experiment 1a, but with double the number of subjects (9 female and 8 male) to obtain sufficient statistical power to assess for potential sex differences in the strength of EtOH conditioning.

*Experiment 1c* also followed the same protocol but employed DBA/2J (*n* = 10) and Swiss Webster (*n* = 6) mice, again using both male and females.

*Experiment 2a* and *2b* were designed to assess the importance of two aspects of the general procedure described in Experiment 1: the every-other-day protocol (2a) and the lack of habituation to the testing procedures before conditioning (2b). For each of these studies we used C57BL/6J male and female mice (8 per group) treated as described above with the exceptions as noted. As always, EtOH-injection day and conditioning context were counterbalanced.

*Experiment 2a* assessed whether the spaced protocol consisting of 2 conditioning days and a test day each separated by a day, could be compressed into a 3 consecutive day paradigm. Here one group was conditioned and tested on consecutive days, while the other half of the subjects received the alternate day exposure and testing as described above.

*Experiment 2b* was conducted to determine whether prior handling and habituation to the test procedures would influence place conditioning. Here we preceded the usual 5-day protocol in half the animals with a 3-day handling procedure during which subjects were removed from the colony room, weighed and injected with i.p. saline in the testing suite, and allowed access to the testing apparatus for 10 min.

### Additional procedural notes

Our general protocol differed in a few ways from those typically employed by researchers studying place conditioning (Cunningham et al., 2006). In addition to the spaced, single-exposure conditioning of otherwise naïve subjects, all subjects were tested at 4–10 h into the dark phase of their light:dark cycle. We don't know whether subjects would condition if they were exposed during the light phase of their cycle because in our lab all behavioral testing occurs during the animals' awake/active phase (they are maintained in a 12:12 reversed light dark cycle with lights off at 09:30). The only other caveat we would add is that our animals' home cage environment was relatively undisturbed during the 5-day protocol, and (as our studies generally began on Monday) perhaps not for the day or two preceding the start of the study. Less robust/more variable results were generated when the corncob bedding was changed during pilot studies.

### Data analysis

Data were analyzed in SPSS 21.0. One sample *t*-tests were carried out to compare time spent in each side of the experimental apparatus on the test day within groups. We evaluated whether the absolute value of the difference between saline time and EtOH time was >0 (or whether the percentage of time spent in the EtOH-paired context was >50%) to assess place preference (or aversion). When comparing two groups we analyzed subjects' behavior using analysis of variance, and in Experiment 1b, with time of testing, in 5 min bins, in a repeated measure analysis to evaluate place conditioning across the testing period.

## Results

### Experiment 1

**Figure 2:**
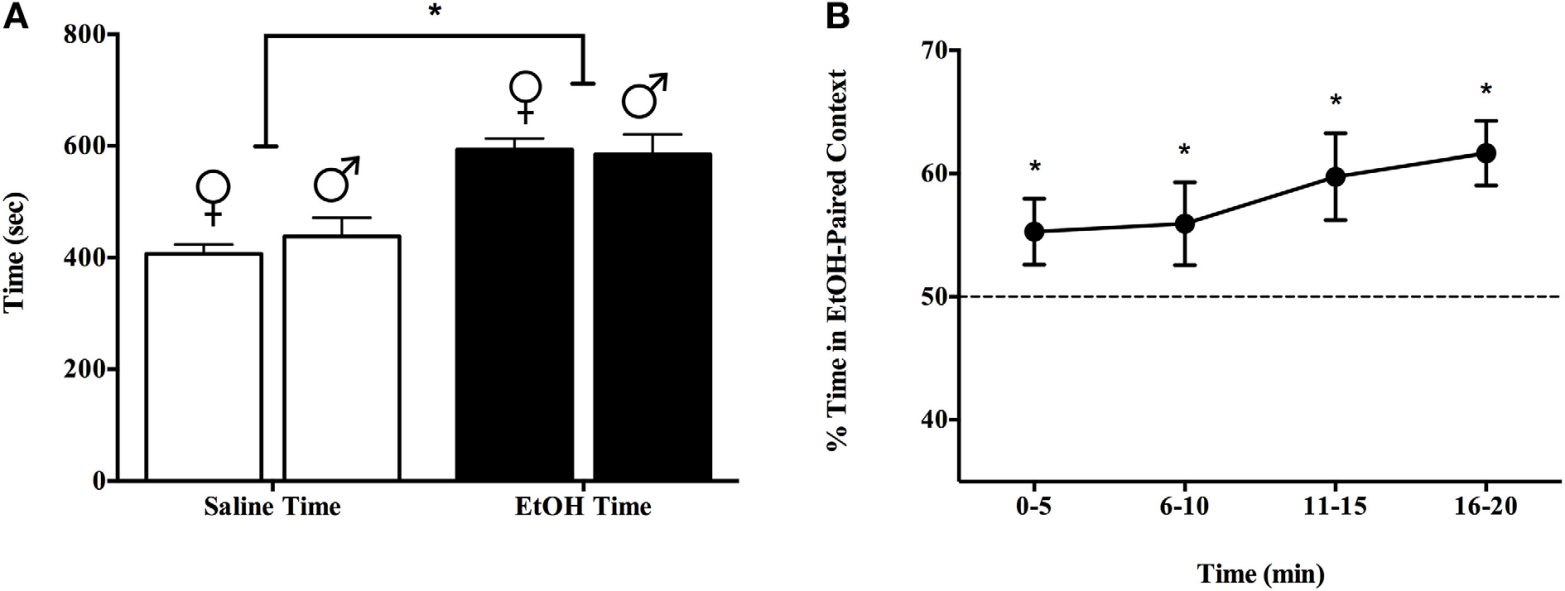
**Experiment 1b demonstrated that both sexes equally prefer a context associated with a single exposure to 1.5 g/kg EtOH over a context paired with saline.** We replicated findings in Experiment 1a (Figure 1) with a larger cohort of male and female C57BL/6J mice (8 male and 9 female naïve, adult subjects). Again, we conditioned and tested over 20 min, on each of days 1, 3, and 5. **(A)** Both male and female subjects spent more time in the EtOH-paired context than in the saline-associated context on day 5 [>57 and 59%, respectively, but did not differ from each other: *F*_(1, 15)_ = 0.30, *p* = 0.592]. **(B)** Preference for the EtOH-paired chamber was evident across the test period and tended to increase with time (from about 55% during the first 5 min to nearly 62% during the last 5 min of testing). ^*^indicates *p* < 0.05.

**Figure 3:**
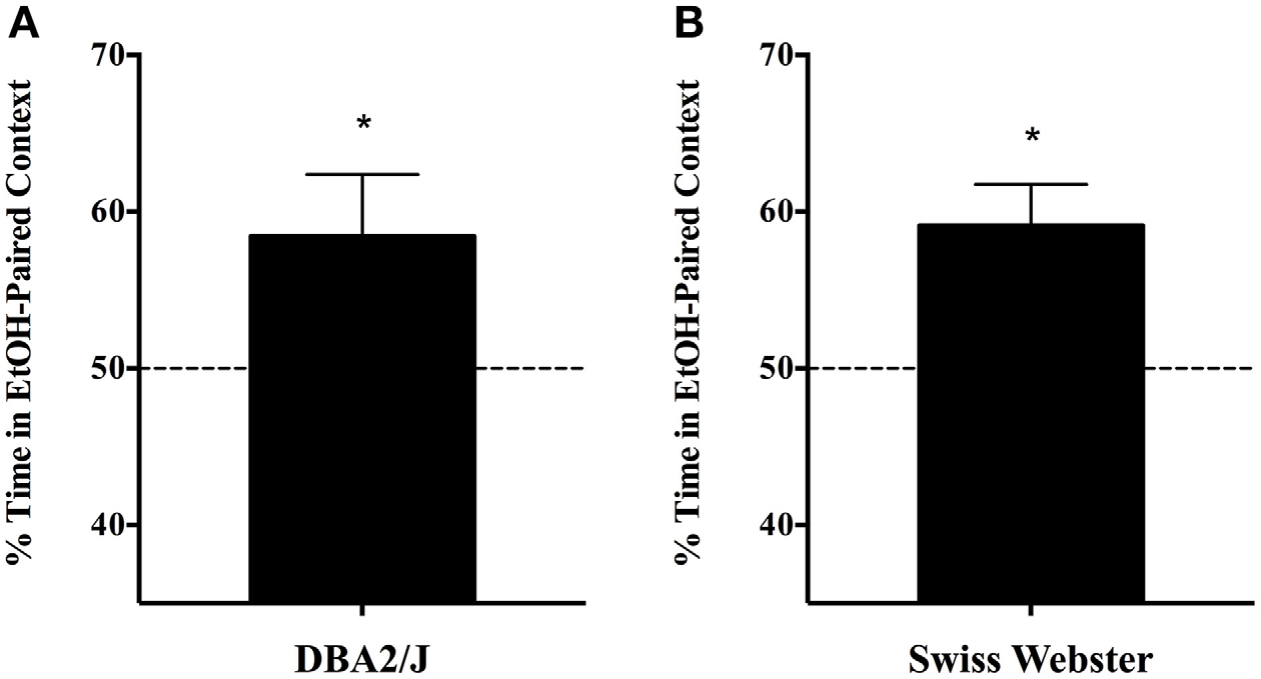
**Single-exposure CPP generalizes to other strains of mice (Experiment 1c). (A)** Male and female naïve adult DBA/2J mice (*n* = 10) were subjected to the standard 5-day, single exposure protocol, and readily developed CPP to the EtOH-paired context [58.4 ± 3.94; mean ± s.e.m.; *t*_(9)_ = 14.804, *p* < 0.001]. Likewise, **(B)** outbred Swiss Webster mice evinced a robust CPP in the single-exposure, spaced protocol [59.1 ± 2.64; *t*_(5)_ = 22.396, *p* < 0.001; *n* = 6]. ^*^indicates *p* < 0.05.

Preference for an environment once paired with 1.5 g/kg EtOH is evident in three strains and both sexes of mice (Figures 1–3). Figures 1C,D show that after receiving 1.5 g/kg EtOH on day 1 or 3 of a 5-day protocol, adult C57BL/6J mice (*n* = 8) preferred the alcohol-paired context to the saline-paired context on day 5. The nearly 2 min difference between the time spent on the sides was significantly greater than zero [*t*_(7)_ = 5.282, *p* < 0.01; Figure 1C]. Subtracting out time spent in the neutral center chamber indicates that on average, subjects spent 57.5% of their onside-time in the EtOH-paired context (±1.46 s.e.m.), as opposed to about 42.5% in the saline-paired environment [two-tailed *t*_(7)_ = 39.362, *p* < 0.001; Figure 1D]. Figure 1E depicts the time spent in the two contexts, which did not differ: *t*_(7)_ = 0.260, *p* > 0.05. It also did not matter whether mice were conditioned to EtOH effects on day 1 or day 3 of the study, as demonstrated by ANOVA *F*_(1, 7)_ = 0.277, *p* > 0.05.

Experiment 1b indicated that the sexes equally prefer a context associated with a single exposure to 1.5 g/kg EtOH over one paired with saline. We replicated findings in Experiment 1a (Figure 1) with a larger cohort of male and female C57BL/6J mice (8 male and 9 female naïve, adult subjects). Again, we conditioned and tested over 20 min, on each of days 1, 3, and 5. Figure 2A shows that both male and female subjects spent more time in the EtOH-paired context than in the saline-associated context on day 5 [>57%, *t*_(7)_ = 2.17, *p* < 0.05] and 59% [*t*_(7)_ = 14.21, *p* < 0.01], respectively, but did not differ from each other: *F*_(1, 15)_ = 0.30, *p* = 0.592. The preference for the EtOH-paired chamber was evident across the test period, non-significantly increasing from about 55% during the first 5 min to nearly 62% during the last 5 min of testing as seen in Figure 2B (all test points were significantly greater than 50% by *t*-test, and the repeated measure ANOVA was >0.05 for time, with no significant interaction).

Single-exposure CPP also generalizes to other strains of mice. Experiment 1c demonstrated that place preference for a context associated with a single, moderate dose of EtOH is also evident in the DBA/2J inbred, as well as outbred Swiss Webster mice. These data are depicted in Figure 3: DBA/2J mice developed CPP to the EtOH-paired context [58.4 ± 3.94; mean ± s.e.m.; two tailed *t*_(9)_ = 14.804, *p* < 0.001; Figure 3A] as did outbred Swiss Webster mice [59.1 ± 2.64; two tailed *t*_(5)_ = 22.396, *p* < 0.001; Figure 3B].

### Experiment 2

**Figure 4:**
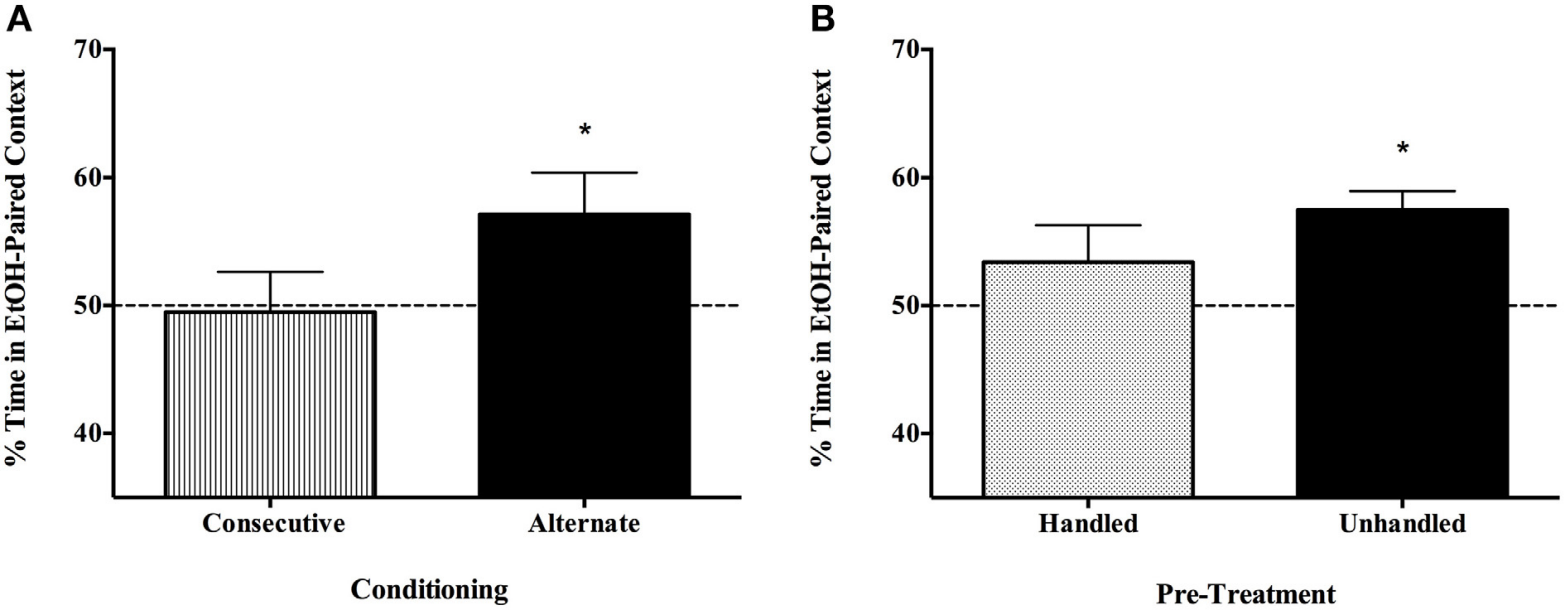
**Place preference is not evident with massed conditioning trials or in subjects that have been habituated to the testing procedures and environment, though spaced conditioning across 5 days in unhabituated/unhandled C57BL/6J subjects yields robust CPP to a single exposure of EtOH. (A)** Conditioning and testing over a 3 day, rather than 5 day period did not produce EtOH place preference. Half of the mice (*n* = 8/group) were administered saline and 1.5 g/kg EtOH in a counterbalanced fashion on day 1 and 2, and tested on day 3 (Consecutive), and the others participated in the alternate conditioning protocol (injections on days 1 and 3; undisturbed days 2 and 4; tested on day 5). Only the Alternate group spent more than 50% of their time in the EtOH-paired context [one-tailed *t*_(7)_ = 2.172, *p* < 0.05]. **(B)** Habituating animals to injections and the testing apparatus before conditioning blocked CPP to EtOH. Only experimentally naïve subjects evidenced place preference [one-tailed *t*_(7)_ = 5.129, *p* < .001; *n* = 8/group]. ^*^indicates *p* < 0.05.

CPP was not evident in a concentrated 3-day protocol where subjects were conditioned on days 1 and 2 and tested on day 3, although again, spaced conditioning and assessment trials reliably resulted in place preference for male and female C57BL/6J mice (Figure 4A). *T*-test confirmed that only the Alternate group spent more than 50% of their time in the EtOH-paired context [one-tailed *t*_(7)_ = 2.172, *p* < 0.05, vs. consecutive *t*_(7)_ = 0.166, *p* > 0.05]. Habituating animals to injections and the testing apparatus before conditioning also attenuated CPP to EtOH (Figure 4B). Only experimentally naïve subjects evidenced place preference [one-tailed *t*_(7)_ = 5.129, *p* < 0.01, vs. handled *t*_(9)_ = 1.168, *p* > 0.05].

## Discussion

Among many factors contributing to alcohol dependence and its constellation of adverse consequences is the subjective experience to the drug. Though no doubt compounded by a host of intervening influences, retrospective reports from alcoholics suggest that early experiences with intoxication are perceived as especially salient and pleasurable (Haertzen et al., 1983; de Wit and Phillips, 2012; Bardo et al., 2013). These reports are in line with the common sense notion that the reinforcing effects of the drug, while varied in the overall population, are especially potent in those at risk for problem drinking. The model described here provides a new and facile tool for investigating the host of influences contributing to this liability in drug naïve subjects, since it assesses subjective rewarding effects of initial drug exposure.

The subjective response to alcohol has been well studied in the clinic since Schuckit first demonstrated that men with a positive family history experienced blunted effects of alcohol compared to men without such a family history (Schuckit, 1980). Over the past few decades, the Low Level of Response Model (LLRM) has been widely investigated, characterizing subjective response as a phenotypic risk factor for alcohol use disorders (Schuckit, 1984; Schuckit and Smith, 1996, 2000, 2001; Schuckit et al., 2007; Trim et al., 2009). Researchers have also looked for, and found, physiological correlates of the LLRM including ataxia (Schuckit, 1985), hormone release (Schuckit et al., 1987a,b, 1988b; Schuckit, 1988) brain activity (Schuckit et al., 1988a; Ehlers and Schuckit, 1991; Trim et al., 2010) and specific molecular markers (Schuckit et al., 1999; Hu et al., 2005; Corbin et al., 2006; Hinckers et al., 2006; Webb et al., 2011).

Despite these many correlations, there is still much that remains to be done in terms of explaining initial sensitivity to the drug and its relationship to future dependence, as well as successfully designing interventions to mitigate a risk-prone phenotype. For example, there are reports contradicting the LLRM, including a number of studies failing to replicate the effect and others finding *greater* response to alcohol in high-risk populations (e.g., Lex et al., 1994; Earleywine, 1995; Moss et al., 2012). Partly because of such contradictions (Newlin and Thomson, 1999), proposed an alternative Differentiator Model (DM), suggesting that future liability is predicted by a “favorable” balance of enhanced positive effects (most prominent on the rising limb of the blood/brain alcohol curve) along with attenuated sensitivity to less reinforcing, sedating effects (evident, especially, as metabolism outpaces absorption) and this idea also has some support (e.g., Martin et al., 1993; King et al., 2002; Erblich et al., 2003; Marczinski et al., 2007). Two groups recently attempted to clarify the issue by meta-analyses (Morean and Corbin, 2010; Quinn and Fromme, 2011) but concur that the extant literature is not definitive, in part because of the variability across clinical studies and populations.

Slow progress in understanding the role of subjective experience on future outcomes can also be attributed to the fact that basic researchers have lacked appropriate animal models for studying the phenotype. Translating the clinical observations to basic studies where experimental control and manipulation can better parse cause and effect will be helpful in testing specific aspects of this broad and complex phenotype, and in turn, inform more targeted approaches in the clinic (Crabbe, 2012).

The assay described here should provide a useful tool for basic researchers since it is a behavioral model of drug reward associated with first exposure. In contrast to previous studies of CPP in rodents, this paradigm does not employ habituation to the conditioning apparatus or procedure, nor does it depend upon repeated EtOH exposure (Cunningham et al., 2006). Drug conditioning and reward assessment (which could be automated) requires only 90 min per subject, a circumstance that would be helpful for high throughput studies. Moreover, the dose used in our experiments (1.5 g/kg) is lower than that typically employed in the place preference model (again, follow Cunningham et al., 2006 for a comprehensive discussion of the CPP protocol). These differences may represent advantages for probing antecedent factors that influence the likelihood of developing drug dependence.

In addition to being entirely conducted during three manipulation sessions across 5 days, conditioned reward to EtOH was robustly demonstrated in three strains of mice (two inbred and one outbred), both sexes, and subject to pharmacological manipulation. The inbred strains employed here, the C57BL/6J and DBA/2J are widely used by researchers investigating addiction. In fact, substantial strain differences in oral consumption of EtOH, first described over 50 years ago (McClearn and Rodgers, 1959) have been used as a basis for a multitude of studies aimed at identifying causes of alcoholism. That the non-preferring DBA strain readily shows CPP to a single dose of EtOH, adds to the growing body of literature suggesting that such strain differences don't mirror differences in the subjective rewarding properties of the drug (Grahame and Cunningham, 1997; Green and Grahame, 2008; Blednov et al., 2012).

Assuming that initial alcohol reward is impacted by multiple genetic loci—a supposition that is widely embraced across the fields of behavioral genetics and pharmacogenetics (Reich et al., 1998; Prescott and Kendler, 1999; Liu et al., 2004) an animal model targeting this intermediate phenotype could help advance the field. The commercial availability of inbred, recombinant inbred, transgenic, and selected lines of mice, presumably evincing a range of responses to this probe, will help elucidate innate factors that contribute to the clinical variation in susceptibility to alcoholism. As with all animal models, value lies in assessing and explaining particular aspects of clinical observations, ultimately proffering targeted interventions for use in humans, and narrowing a translational gap.

Additional factors, beside genetic, might also be fruitfully investigated. For instance, selective antagonists might be used to assess neurochemical contributions such as those from endogenous opioids or other peptides (Froehlich et al., 2000; Gianoulakis, 2009; Walker et al., 2012; Ubaldi et al., 2013), as well as classical or novel neurotransmitters and their receptors (Pava and Woodward, 2012; Trudell et al., 2014). Moreover, developmental and other environmental manipulations (e.g., stress, housing conditions; Clarke et al., 2012) can be readily evaluated in a single-exposure CPP paradigm.

It remains to be determined whether or not a single exposure to other reinforcing substances will lead to a CPP. It may be that some, but not all abused drugs are reinforcing from the start, while others require neuroadaptation to mediate subjective reward. The fact that CPP in our model was attenuated by prior habituation and handling, for instance, could suggest that the behavioral changes indicative of initial subjective reward to alcohol depend upon the novelty of the context. If so, the subjective reward we demonstrated might result more from negative, than positive, reinforcement. If so, sedative-anxiolytic drugs might also be reinforcing in a model such as ours, while reward from other classes of drugs, such as stimulants, might benefit from habituation prior to conditioning and/or multiple drug exposures. Novel environments are considered mildly stressful, and in such contexts the anxiolytic effects of alcohol might be experienced as especially reinforcing. If so, more anxious subjects (either by “nature” or “nurture”) should demonstrate stronger CPP (Dockstader and van der Kooy, 2001), and could be useful in understanding the relationship between anxiety disorders and alcohol use disorders that has been well substantiated in the clinic (e.g., Kushner et al., 2005; Brady et al., 2007; Farris et al., 2012).

Worldwide, addiction may be the most formidable health problem, affecting about 1 in every 5 people over the age of 14 (World Health Organization, 2011). Though alcoholism, like other complex heterogenous traits, results from a dense constellation of interacting influences, a better understanding of the initial subjective rewarding effects will facilitate theory-driven approaches to treating this devastating disorder. The single-exposure CPP to alcohol demonstrated in our studies provides a new tool for investigating factors that predict disordered drinking. Because at least some of the antecedent factors are present before, and evident upon, first exposure to the drug, interventions and treatments for the disease critically depend upon better understanding the vulnerable predisposition.

### Conflict of interest statement

The authors declare that the research was conducted in the absence of any commercial or financial relationships that could be construed as a potential conflict of interest.
